# Tunable Low Crystallinity Carbon Nanotubes/Silicon Schottky Junction Arrays and Their Potential Application for Gas Sensing

**DOI:** 10.3390/nano11113040

**Published:** 2021-11-12

**Authors:** Alvaro R. Adrian, Daniel Cerda, Leunam Fernández-Izquierdo, Rodrigo A. Segura, José Antonio García-Merino, Samuel A. Hevia

**Affiliations:** 1 Instituto de Física, Pontificia Universidad Católica de Chile, Casilla 306, Santiago 6904411, Chile; aradrian@uc.cl (A.R.A.); ddcerda@uc.cl (D.C.); jose.garcia@uc.cl (J.A.G.-M.); 2 Centro de Investigación en Nanotecnología y Materiales Avanzados, Pontificia Universidad Católica de Chile, Casilla 306, Santiago 6904411, Chile; 3 Department of Material Science & Engineering, The University of Texas at Dallas, Richardson, TX 75080, USA; lxf180007@utdallas.edu; 4 Instituto de Química y Bioquímica, Universidad de Valparaíso, Avenida Gran Bretaña 1111, Valparaíso 2340000, Chile; rodrigo.segura@uv.cl

**Keywords:** low crystallinity carbon nanotubes, anodic aluminum oxide, electric transport, gas sensor, Schottky junction arrays

## Abstract

Highly ordered nanostructure arrays have attracted wide attention due to their wide range of applicability, particularly in fabricating devices containing scalable and controllable junctions. In this work, highly ordered carbon nanotube (CNT) arrays grown directly on Si substrates were fabricated, and their electronic transport properties as a function of wall thickness were explored. The CNTs were synthesized by chemical vapor deposition inside porous alumina membranes, previously fabricated on n-type Si substrates. The morphology of the CNTs, controlled by the synthesis parameters, was characterized by electron microscopies and Raman spectroscopy, revealing that CNTs exhibit low crystallinity (LC). A study of conductance as a function of temperature indicated that the dominant electric transport mechanism is the 3D variable range hopping. The electrical transport explored by I–V curves was approached by an equivalent circuit based on a Schottky diode and resistances related to the morphology of the nanotubes. These junction arrays can be applied in several fields, particularly in this work we explored their performance in gas sensing mode and found a fast and reliable resistive response at room temperature in devices containing LC-CNTs with wall thickness between 0.4 nm and 1.1 nm.

## 1. Introduction

Carbon nanotubes (CNTs) remain being considered as promising materials in science and technology owing to their multiple outstanding properties [1,2,3,4]. However, these properties strongly depend on the crystallinity or graphitization level of their walls since this determines the electronic structure [5], and consequently, the optical [6], electric [7], and mechanical [8] properties. These nanostructures have proved to be useful as active materials in photo-actuators [9], photodetectors [10], semiconductor electronics [11], and gas sensors [12]. Nevertheless, it is still challenging to produce an array of CNTs with the same morphological characteristics and, therefore, the same physical properties [13]. A particularly suitable method for growing highly ordered nanotube arrays is to use an anodic aluminum oxide (AAO) as a template. One of the significant advantages of using AAO membranes is the cost-efficient synthesis of large area arrays of densely packed nanopores with well-controlled dimensions in the nanometer range. Also, using this dielectric matrix to deposit the carbon nanostructures provides mechanical support to fabricate robust devices [14]. Using the chemical vapor deposition (CVD) process, the diameter, length, wall thickness, and graphitization level of CNT can be controlled [15,16,17,18]. The controllability in morphology has an advantage in tuning physical properties [19,20]. The use of these arrays has been reported in electronic systems with better performance than those using non-organized CNTs [21,22]. However, the synthesis of millions of CNTs will hardly be completely crystalline, and there will be amorphous components that affect specific properties. Therein lies the importance of studying and classifying the effects of systems with a high degree of disorder.

The distinctive feature that low-crystalline materials exhibit is that they have localized electronic states; therefore, in a material with a strong structural disorder, an electronic structure with highly localized states arises, exhibiting an electric transport mechanism known as variable range hopping (VRH) [23]. In this case, the conductance tends to zero as the temperature tends to zero due to the hopping process is a phonon-assisted mechanism that transfers an electron from one localized state to another [24]. A good description of this mechanism opens the possibility to tailor the global transport properties in arrays with highly structural defects [25]. The contribution to study CNTs with localized states has a relevant impact since macroscopic samples generally contain several distortions, either in the diameters, chirality, or doping [26]. In this case, rather than making an individualized picture of the properties of the nanotubes, it is necessary to generate approximate models of the average characteristics. For that reason, this work analyzes the electric transport in low-crystallinity CNTs (LC-CNTs) with controllable dimensions in a robust device consisting of Si substrates.

The heterojunction form between carbon allotropes and Si has been reported as a Schottky junction, in which the carrier transport generally follows the thermionic emission (TE) theory at room temperature [27,28,29]. Moreover, other conduction mechanisms are superposed in complex nanosystems interfaces such as space-charge limited or tunneling [30]. These components are difficult to explore due to several variables, such as substrate doping, CNTs/Si contact, or temperature [31]. It is important to approach the heterojunction as a global system and isolate the crystalline and non-crystalline components from CNTs. For instance, the study of CNTs and Si junctions using AAO and CVD process with synthesis temperature of 950 °C has been reported [31,32,33]. At this temperature, the CNTs start to lose the defects and behave more as multiwall CNTs with metallic properties [34]. The effects that the impurities in the crystal lattice can induce in electronic transport are not observed in these systems. For that reason, CNTs synthesized at low temperatures are expected to present low crystallinity, and therefore, the effects of interest in the electronic transport mechanism can be observed. Moreover, it is important to analyze the behavior as a function of wall thickness and how this affects the device junction due to dimensionality effects. Furthermore, since these heterojunction devices have been proved to sense gases [34,35], it is appropriate to explore this characteristic in the proposed LC-CNTs. The tunability of electrical transport will allow exploring different morphologies to optimize gas sensing.

This paper presents the synthesis, characterization, and study of the electrical transport properties of LC-CNTs arrays grown directly on Si substrates using AAO as a template. The dominant electric transport mechanism in LC-CNTs is the 3D-VRH and shows a strong dependence on the wall thickness of tubes. Moreover, the LC-CNTs arrays are exposed to reducing gases, and they exhibit a dependence of its electric resistance as a function of gas concentration, which opens the possibilities to use it as gas sensors.

## 2. Materials and Methods

### 2.1. Synthesis of Porous Alumina Membranes on Silicon Substrates

Porous alumina membranes (PAMs) were fabricated on Si substrates using a 5 µm layer of Al (99.999% purity). The Al was deposited at a rate of 2 Å/s over polished n-type Si (100) wafers (1–10 Ω∙cm) by electron beam evaporation. The Al film was anodized in two steps to obtain PAMs with highly ordered pore patterns [36]. The first anodization step was performed at 40 V in 0.3 M oxalic acid for a period of 40 min, maintaining the temperature of the electrolyte at 5 °C. Furthermore, an aqueous solution with 6.0 wt% phosphoric acid and 1.8 wt% chromic acid at 60 °C was employed to remove the porous alumina layer produced in the first anodization step, leaving an ordered pattern of pores nucleus in the surface of the mask. Then, the second anodization was performed under the same previously mentioned conditions for 50 min, yielding a homogeneous and ordered membrane with a thickness close to 2.5 µm. Finally, to remove the alumina barrier layer at the bottom of the pores and widen the pores without affecting the membrane order, the samples were subjected to an etching treatment with a 5 wt% phosphoric acid solution at 21 °C for 55 min.

### 2.2. Low Crystallinity Carbon Nanotubes Synthesis

The synthesis of LC-CNTs inside the pores of PAMs was achieved by CVD method, using a horizontal tube furnace (MTI - OTF 1200X furnace, MTI Corp., Richmond, CA, USA). A piece of PAM/Si (~1 cm^2^) was heated at the center of the reactor at a rate of 30 °C/min under an Ar atmosphere (200 sccm) until reaching 650 °C. Then, a flow of C_2_H_2_ at 25 sccm was introduced as a carbon source, and CNTs were synthesized at different times: from 1 to 30 min. This process promotes the growth of controllable nanotubes inside the pores with tunable wall thickness by keeping the temperature constant [36]. Finally, the whole system LC-CNTs/PAM/Si was cooled down to 21 °C under Ar environment.

### 2.3. Deposition of the Top Electrode

Top electrodes, consisting of a ~10 mm^2^ circular area of 99.9% pure Au, were deposited perpendicular to the top of the LC-CNT/PAM/Si samples at an evaporation rate of 0.4 Å/s until obtaining 100 nm of Au thickness. The deposition was performed in a Balzers evaporator equipped with a Temescal STIH-270-2PT (Ferrotec, Livermore, CA, USA) electron beam source operated at 8 kV and a quartz crystal microbalance to measure the evaporation rate and deposited thickness.

### 2.4. Characterizations

The structural characterization was carried out through Raman Spectroscopy using a LabRam010 spectrometer (Horiba. Ltd., Kyoto, Japan) at 632.5 nm wavelength. Scanning electron microscopy (SEM) and transmission electron microscopy (TEM) were used to characterize the samples morphologically. SEM analysis was carried out using a FEI Quanta 250 FEG (Thermo Fisher Scientific, Waltham, MA, USA). Standard TEM analysis was performed using a Hitachi HT7700 (Hitachi High Tech Co., Ltd., Tokyo, Japan), and high-resolution TEM (HR-TEM) by using a FEI Tecnai ST-F20 microscope (FEI Company, Hillsboro, OR, USA). In order to perform the TEM measurements, CNTs were released from the PAM by dissolving it in sodium hydroxide solution 3.5 M at 21 °C. Afterward, samples were rinsed with double distilled water and suspended in isopropanol alcohol to obtain a CNTs dispersion.

### 2.5. Electric Transport Measurements as a Function of Temperature

The electric transport properties were studied in the samples as a function of the wall thickness by analyzing the I–V curves. This measurement assists in the identification of the existing junction between the Si substrate and the LC-CNTs. The I–V curves were measured in the voltage range from −1.5 to 0.8 V to predict a model which fits the electrical characteristics of the junction. Moreover, the conductance was measured around zero bias as a function of temperature from 10 to 300 K to study the dominant transport mechanism. The samples were biased by contacting the top Au-electrode and the Si substrate. The measurements were performed with a Keithley electrometer model 6517B (Keithley Instruments Inc., Cleveland, OH, USA) in a 10 K closed cycle refrigerator system from Janis Research Company (Wilmington, MA, USA) with high vacuum conditions (<10^−6^ Torr). The resistance of the fabricated devices is several orders of magnitude larger than the total resistance of the wires and electrodes; therefore, the errors introduced by using a two-probe measurement are negligible.

### 2.6. Room Temperature Resistance Measurements in Different Atmospheres

The electrical response of the samples was analyzed in a perturbed atmosphere to test their performances as gas sensors. These were exposed to different cycles of H_2_ and C_2_H_2_ concentrations under an Ar atmosphere at room temperature (21 ± 1 °C), atmospheric pressure, and absence of light. The resistive response (S), shows in Equation (1), is defined as the percentage change in electric resistance when the device is exposed to the analyte (*R*(*t*)) compared to Ar environment (*R_Ar_*). The electric resistance was measured using a Keithley 6487 picoammeter (Tektronix, Portland (Beaverton), OR, USA) around zero bias.

(1) $$S=\frac{R(t)-R_{Ar}}{R_{Ar}}100\%$$

The gas mixtures were prepared on the base of a total flow of 100 sccm controlled by Alicat (Alicat Scientific Inc., Tucson, AZ, USA) Mass Flow Controllers (Models MC-5SLM for Ar and MC-10SCCM for H_2_ and C_2_H_2_). The measured response corresponded to changes in current when the device was exposed to a certain amount of analyte by a certain amount of time.

## 3. Results and discussions

### 3.1. Morphological and Structural Characterization

**Figure 1:**
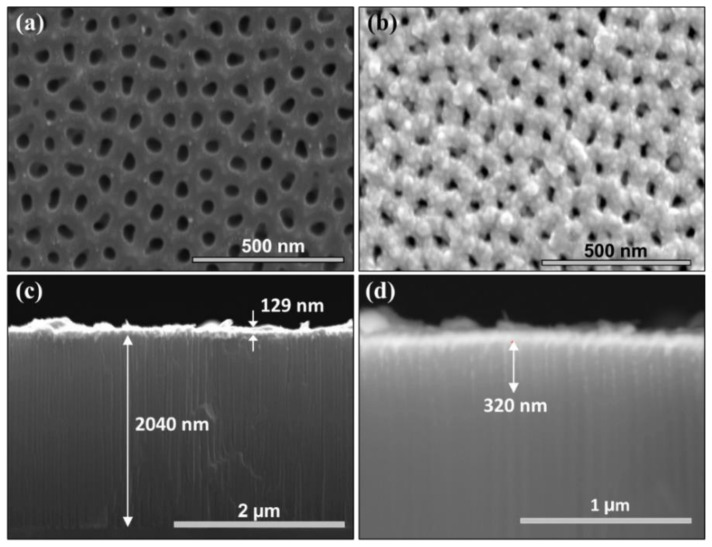
SEM micrographs of a sample synthesized with 5 min of growth time. (**a**) Top view of the pristine sample. (**b**) top view after Au deposition. (**c**) Side view, showing the height of the PAM and the thickness of Au-electrode. (**d**) Backscattered electron image side view, showing the Au penetration inside the nanopores.

Figure 1 shows SEM micrographs of a LC-CNTs/PAM/Si sample, synthesized with 5 min. Figure 1a corresponds to a top view of a PAM with the LC-CNTs grown inside, and Figure 1b shows the same sample after the Au deposition. The incorporation of the top electrode keeps the pores open, but their average diameter is reduced from 43 ± 6 nm to 30 ± 9 nm. Figure 1c shows a lateral view of the sample, in which the electrode thickness (129 ± 23 nm) and the PAM height (2040 ± 2 nm) are determined. Since the Au is deposited with the sample oriented perpendicular to the evaporated metal flow, the Au partially penetrates the nanotubes. Figure 1d shows a backscattered electron image which evidences that the Au average penetration inside the pores is about 320 ± 65 nm. Thus, the Au and Si-electrodes are electrically connected only through the LC-CNTs.

**Figure 2:**
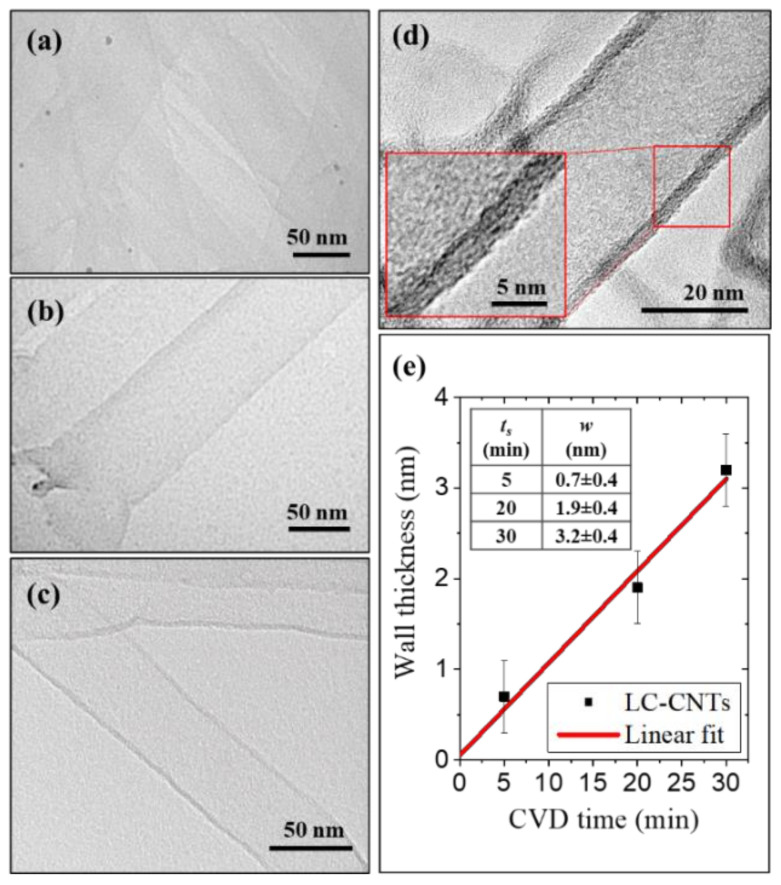
TEM micrographs of LC-CNTs growth with 25 sccm C_2_H_2_ flow at (**a**) 5 min, (**b**) 20 min, and (**c**) 30 min of synthesis time. (**d**) HR-TEM micrograph of sample growth with 30 min of synthesis time. (**e**) Wall thickness as a function of the time synthesis plot obtained from the HR-TEM images.

Figure 2a–c show TEM images of the LC-CNTs grown with 5, 20, and 30 min of synthesis time, respectively. Moreover, Figure 2d corresponds to a representative HR-TEM image of a nanotube synthesized at 30 min. From this image, it is possible to notice the low degree of crystallinity of the CNTs. The average wall thickness of LC-CNTs with 5 min, 20 min, and 30 min of synthesis time is 0.7 ± 0.4 nm, 1.9 ± 0.4 nm, and 3.2 ± 0.4 nm, respectively. These average wall thicknesses are uniform along the vertical direction of the CNTs. The thickness (w) as a function of time synthesis (ts) is plotted in Figure 2e, and a linear dependence is observed.

**Figure 3:**
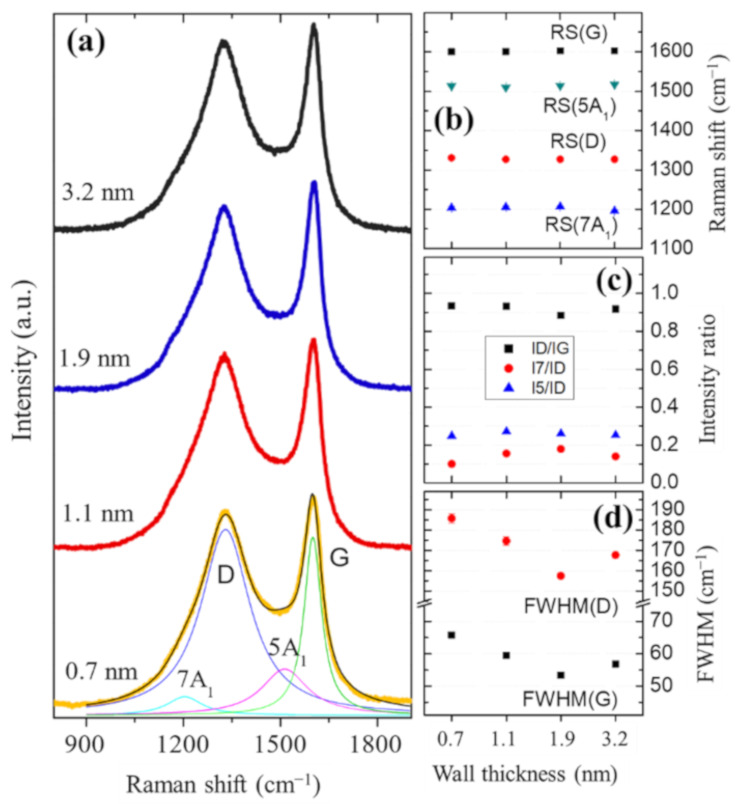
(**a**) Raman spectra of LC-CNTs as a function of wall thickness. (**b**) Peak position of resonances 7A_1_, D, 5A_1_, G. (**c**) Representative ratio of I(D)/I(G), I(7A_1_)/I(D), and I(5A_1_)/I(D). (**d**) FWHM of G and D peaks.

**Figure 4:**
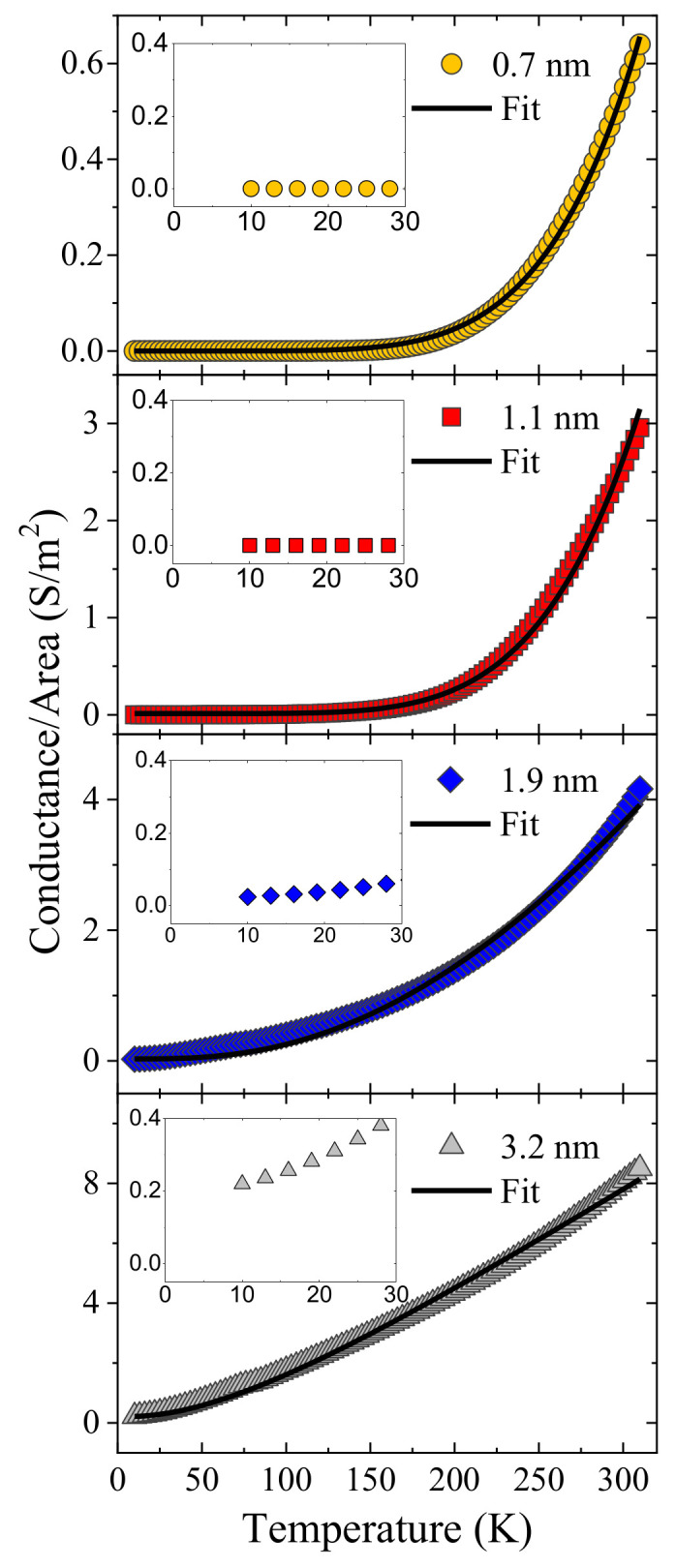
Conductance as a function of temperature and wall thickness of LC-CNTs. Insets show a zoom near 10 K.

Figure 3 shows the first-order Raman spectra between 850 cm^−1^ and 1900 cm^−1^ range of samples with a wall thickness of 0.7 nm, 1.1 nm, 1.9 nm, and 3.2 nm. The spectra showed two main resonances located around 1326 cm^−1^ and 1600 cm^−1^, which correspond to the G and D bands of carbonaceous materials, respectively [37]. Both resonances are linked to vibrational modes of sp^2^ bonded carbon atoms. The G peak involves the bond-stretching motion of C–C atoms (E_2g_ vibrational mode), which occurs even without the presence of the six-fold aromatic rings [38]. The D resonance can be linked to an active A_1g_ breathing mode in amorphous carbon structures [39,40]. In this case, the spectra are characteristic of carbon nanotubes with a low degree of graphitization [37]. Besides, two peaks labeled as 7A_1_ and 5A_1_ shown in Figure 4a need to be considered to fit the data. These resonances, located around 1200 cm^−1^ and 1510 cm^−1^, can be attributed to the breathing modes of seven and five-member carbon rings, respectively [41].

From the Raman spectra and the plots of fit parameters shown in Figure 3, it is possible to realize that the degree of graphitization of the LC-CNTs does not exhibit significant changes concerning the wall thickness. In all the samples, the Raman shift position of the four resonances, RS(7A_1_), RS(D), RS(5A_1_), and RS(G), is almost constant. Similar behavior is observed for intensity ratios between the peaks, and particularly in the case of D and G peaks, in which the intensity ratio has a value close to 0.9. On the other hand, the full width at half maximum (FWHM) of G and D peaks (FWHM(G) and FWHM(D)) tend to decrease in a bounded range. These values are an indication that the samples have a similar level of graphitization. The low crystallinity of the CNTs observed in TEM micrographs and Raman spectra agree with previous work [15,22,42].

### 3.2. Study of Conductance as a Function of Temperature

Since there is a considerable degree of disorder in the studied CNTs, a conductance analysis as a function of temperature was used to determine their electric transport properties. Figure 4 shows the conductance characterization of the same samples analyzed by Raman spectroscopy. The electrical behavior of all LC-CNTs exhibits a non-metallic temperature dependence, which can be mainly explained by using the variable range hopping (VRH) model [23]. This transport promotes that charge carriers move by phonon-assisted hopping between localized states. The conductance at zero electric fields can be obtained by Mott’s law [43] as follows:

(2) $$G=G_{h}\exp\left[-{\left(T_{0}/T\right)}^{1/\left(d+1\right)}\right]$$

where *G_h_* is the hopping conductance, *T* is the absolute temperature, *T*_0_ = *α*^3^/*k_B_n*(*E_f_*) is the characteristic activation temperature and is a measure of the degree of electronic localization, which depends on the parameter *α* that is related to the spatial decay of the localized electronic state, *n*(*E_f_*), the density of localized electronic states at the Fermi level, and the Boltzmann constant (*k_B_*). The dimensionality value is *d* = 3, obtained from the best fit of the data, which indicates that the dominant electric transport mechanism is the three-dimensional variable range hopping (3D-VRH). However, as the LC-CNT wall thickness increase, the conductance does not tend to zero when the temperature tends to zero (see insets in Figure 4), as is required by Equation (2). Hence, it is necessary to add a *G_m_* parameter that can be considered as roughly independent of temperature, and which represents the main contribution of a metallic transport mechanism acting in parallel to the 3D-VRH for the low-temperature range (<20 K). It is important to mention that some other transport mechanisms could explain this non-phonon assisted conductivity, such as Bloch Grüneisen [44] or FIT [45]. However, the contribution of *G_m_* is at least two orders of magnitude lower than VRH around 300 K, therefore, for simplicity, the model with the fewest free fit parameters was used for the electric conductance curve fitting. The parameters of the fitting of each curve are presented in Table 1. It is noticed that *T*_0_ decreases when the wall thickness increases. This value changes up to two orders of magnitude from 0.7 nm to 3.2 nm of wall thickness and is in good range for amorphous systems (10^5^–10^12^ K) [46]. This difference could be originated by an increment in *n*(*E_f_*) or a reduction of the localized electronic wave function *α*. Nevertheless, the magnitude of this difference is expected to mainly arrive from a change in *α*, due to *T*_0_ has a cubic dependence on this parameter. The previous discussion implies that the wave functions are less localized as the wall thickness increases, which is also consistent with the observed behavior of the parameter *G_m_*.

### 3.3. Gas Sensing Measurements

**Figure 5:**
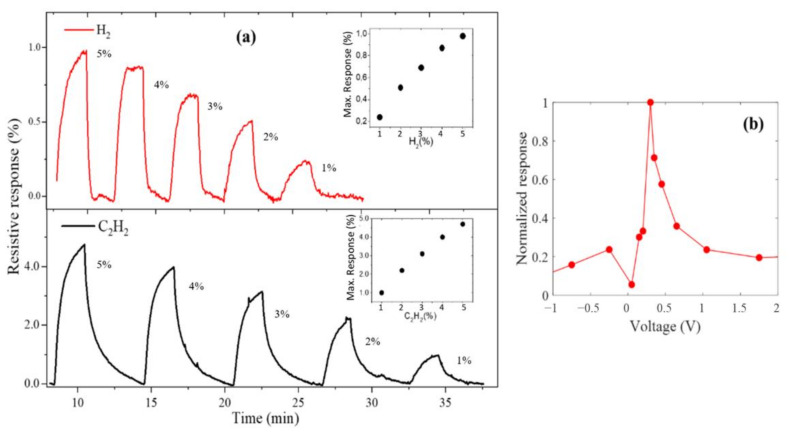
(**a**) Gas sensing behavior of sample with 0.7 nm wall thickness at different H_2_ and C_2_H_2_ concentrations. Insets show the maximum sensitivity percentage as a function of the analyte concentration. (**b**) Normalized resistive response as a function of bias voltage.

The tunable electric transport exhibited by the nanotubes as a function of wall thickness opens the possibility to design devices for a particular application. For instance, a sensor to detect a certain gas atmosphere is feasible since the molecules interacting with the sample are expected to change the electrical parameters [47]. To study this principle, an experiment to measure the electrical resistance was performed in a gas chamber for a set of samples with wall thickness between 0.3 to 3.2 nm. Figure 5a shows the resistive response of a specific device with 0.7 nm wall thickness under different concentrations of analytes (H_2_ and C_2_H_2_) from 1% to 5%. It is observed five representative cycles of the transient responses under H_2_ and C_2_H_2_ analytes. The maximum values reveal a quasi-linear dependence on both gas concentrations (insets), which is an indication that the sensing mechanism is not saturated under these conditions. The resistive response has been tested in different bias voltages, and the results are exposed in Figure 5b. For both analytes, the sample exhibits their maximum response at biased closed to 0.3 V. This nonlinear behavior is consistent with the observed in materials forming junctions in which the maximum sensitivity is related to a potential barrier [48].

Additionally, the resistive response of several devices was evaluated at the same concentrations of H_2_ and C_2_H_2_, and a bias voltage around 0.3 V. Particularly, a strong dependence as a function of the wall thicknesses of LC-CNTs was observed. Table 2 summarizes the maximum response measured under H_2_ and C_2_H_2_, and the conductance at zero bias. Only the devices containing LC-CNTs with wall thicknesses between 0.4 and 1.9 nm exhibit a response to the presence of the analytes. In the case of devices with thin walls (<0.4 nm), a high noise was presented in the current measurements; since thinner tubes have a very low conductance (bellow 5 × 10^−3^ S/m^2^), the gas sensing signal was overlapped to the noise. Moreover, for tubes with thicker walls (>1.1 nm) and higher conductance, the sensibility tens to disappear. For both analytes and in all tested concentrations, the response time of the arrays was less than 15 s, a period that can be mainly attributed to the time the analyte takes flowing from the flow controller to the device. The half-maximum time, period which the sensor takes to reach half of the maximum response, was observed between 15 s and 25 s for all cases, being just a few seconds faster for H_2_ than C_2_H_2_.

Previous reports indicate that devices built on the same conditions as these LC-CNTs do not respond electrically to H_2_ perturbation by a reaction effect [35]. Besides, the results of Figure 5b point out that the LC-CNTs/Si samples contain a diode-like junction since the electrical response is more representative around 0.3 V bias voltage. This effect is common in porous nanomaterials in which the gas produces a perturbation at the junction interface that gives rise to a change in the electrical signal [49,50,51,52]. The physical mechanism behind this is that the gases permeate through the pores until it reaches the contact barriers, producing the electrical difference. Moreover, we performed the gas sensing experiment in self-supported LC-CNTs (without the Si substrate), using Au electrodes in the top and the bottom, and there was no change in the resistivity response. Thus, the heterojunction is expected to promote the sensing gases mechanism and, to explore its nature, an analysis of the electrical transport is carried out below.

### 3.4. Electrical Characterization of the LC-CNTs/Si Junction

**Figure 6:**
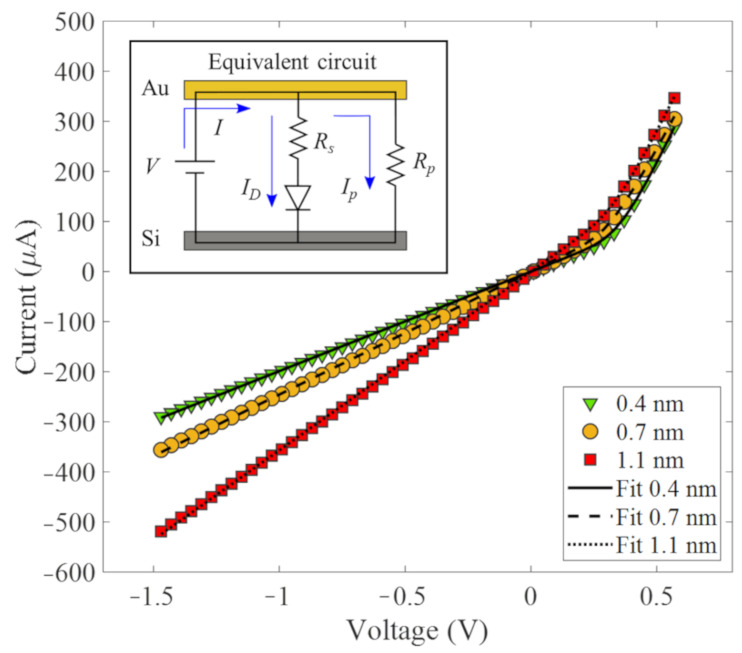
Room temperature dark I–V curves of samples with wall thickness of 0.4 nm, 0.7 nm, and 1.1 nm. Equivalent circuit (inset).

To explain the previous results, we study the junction between LC-CNTs and Si, with a focus on the devices that exhibit the highest sensitivity. For that purpose, the dark I–V curves were measured on samples that contain LC-CNTs with 0.4 nm, 0.7 nm, and 1.1 nm of wall thickness, in a voltage range from −1.5 V to 0.7 V at room temperature, connecting the positive terminal to the LC-CNTs (Au-electrode) and the negative to the Si substrate. The results are plotted in Figure 6, in which it is observed that the curves exhibit a rectifying behavior with a high reverse current. In fact, these I–V curves can be modeled by an equivalent circuit consisting of a resistance connecting in parallel with a diode and a resistance in series, as shown in the inset of Figure 6.

The I–V curves indicate that the arrays present two kinds of junctions attributed to the contacts between LC-CNTs and Si. Since below 0 V, the I–V curves have linear behavior, and above 0 V has a nonlinear tendance, the simplest model that explains this behavior is a parallel system composed of rectifying (Schottky) and non-rectifying (ohmic) junctions. The resistance in parallel (*R_p_*) of the circuit represents the equivalent resistance of all LC-CNTs ohmic-connected with the substrate. Meanwhile, the resistance in series (*R_s_*) represents the equivalent resistance of the LC-CNTs connected to the Si that forming a rectifying junction. Thus, *R_s_* considers the resistance of the LC-CNTs plus the junction resistances. This junction can be modeled as a Schottky barrier [31,53], and the current through the thermionic emission diffusion (TED) theory [54], described by:

(3) $$I_{D}=I_{s}\left[\exp\left(\frac{V}{nV_{T}}\right)-1\right]$$

This model initially developed for charge carrier transport across potential barriers in crystalline materials has also been used for non-crystalline systems [49]. Considering the equivalent circuit (inset Figure 6), the total current through it is given by the expression [55]:

(4) $$I=I_{D}+I_{p}=I_{s}\left[\exp\left(\frac{V\left(1+R_{s}/R_{p}\right)-IR_{s}}{nV_{T}}\right)-1\right]+\frac{V}{R_{p}}$$

where *I_D_* is the current through the diode and *R_s_*, *I_p_* is the current through *R_p_*, *I_s_* corresponds to the saturation current, *n* is the ideality factor, and *V_T_* = *k_B_T* is the thermal voltage. The analytical solution of this equation can be obtained using the Lambert *W* function [55].

(5) $$I=\frac{nV_{T}}{R_{s}}W\left\{\frac{I_{s}R_{s}}{nV_{T}}\exp\left(\frac{V+I_{s}R_{s}}{nV_{T}}\right)\right\}-I_{s}+\frac{V}{R_{p}}$$

Since the model is dictated by thermionic emission, *I_s_* has the following expression:

(6) $$I_{s}^{}=A\cdot A^{*}\cdot T^{2}\cdot \exp\left(\frac{-\phi_{B}}{V_{T}}\right)$$

Where *A* is the contact area, A* is the Richardson constant, and *ϕ_B_* is the barrier voltage.

Table 3 shows the parameters of the I–V curves fitting of Figure 6 and other relevant information calculated from the fit parameters. It is observed that n, which is a measure of conformity of the diode behavior to TED theory, has a close value to 1 (ideality), indicating that the model is appropriate to describe the charge transport across the junction. The equivalent resistance *R_p_* decreases as the wall of the CNTs widens. This dependence was expected due to his value is related to the individual resistance of the nanotubes, which are less resistive as their wall thickness increases. The value of *R_s_* slowly decreases as the wall widens; however, in this case, it is not possible to attribute this only to the resistance of the LC-CNTs due the junction resistances are expected to contribute to the equivalent resistance Rs. However, the values of *R_s_* and *R_p_* can be used to get a lower bound of the percentage of CNTs connected through a Schottky junction (*SC*), because *R_s_* is an upper bound of the equivalent resistance of the CNTs connected through a Schottky junction but without considering the junction resistance contribution. This percentage is given by *SC* = 100/(1 + *R_s_*/*R_p_*), and as we can be observed in Table 3, the number of CNTs connected through a Schottky junction is more than 80% in all the cases.

From the currents *I_s_*, whose values are according to the literature [31,56], and based on Equation (6), it is possible to estimate an average barrier voltage *ϕ_B_* of the junctions. For that, first is necessary to compute the contact area *A* for each sample. Considering that one nanotube has a cross-sectional area given by the expression *A_CNT_* = *wπ*(*D_p_* − *w*), where *w* is wall thickness, and *D_p_* ≈ 50 nm is the average pore diameter of the bare AAO, is obtain a values of about 6 × 10^−17^ m^2^, 11 × 10^−17^ m^2^, and 17 × 10^−17^ m^2^ for samples with 0.4 nm, 0.7 nm, and 1.1 nm wall thickness, respectively. Then, the total number of CNTs connected in each sample is around 1 × 10^9^, since the area of the top electrode is ~10 mm^2^ and the pores cover about 20% of the total area of the electrode (determined from an image processing method of a SEM picture of a bare PAM). Finally, the total contact area A of the Schottky junctions can be calculated considering the percentage of the CNTs connected to the Si through this junction. The values for *A* are shown in Table 3, together with the average barrier voltage *ϕ_B_*, which has values between 0.34 to 0.36 eV for the three samples.

**Figure 7:**
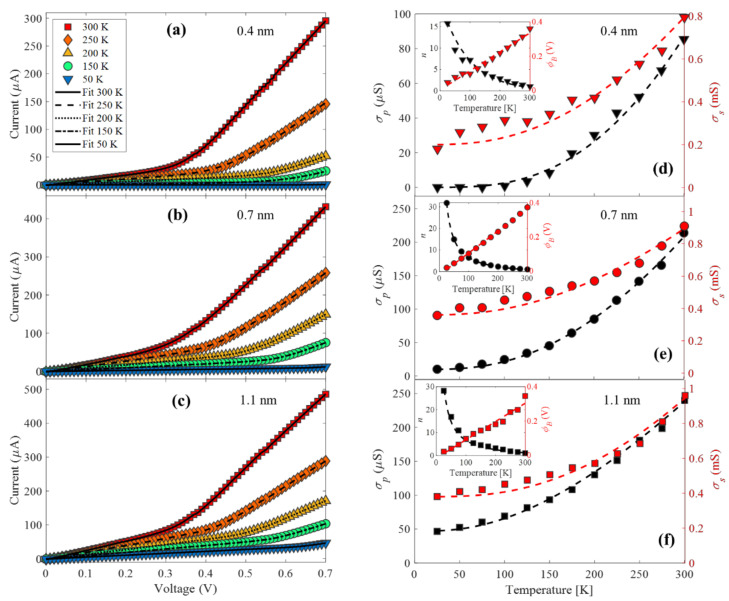
(**a**–**c**) I–V curves measured at 300 K, 250 K, 200 K, 150 K, and 50 K of samples that contain LC-CNTs with 0.4 nm, 0.7 nm, and 1.1 nm of wall thickness, respectively. (**d**–**f**) are the plot of the conductance *σ_p_* = 1/*R_p_* and *σ_s_* = 1/*R_s_* as a function of temperature for each sample, the insert shows the ideality factor and the average barrier voltage *ϕ_B_* as a function of temperature.

Figure 7a–c shows the dark I–V curves as a function of temperature (20 K to 300 K) of samples that contain LC-CNTs with a wall thickness of 0.4 nm, 0.7 nm, and 1.1 nm, respectively. The three samples exhibit the same behavior: as the temperature decrease, the slope of both sides of the I–V curves decreases, and the region voltage in which the curve experiences the change in the slope moves to a higher voltage. With the model previously discussed, we fit the I–V curves over the full range of temperatures. The conductance *σ_p_* = 1/*R_p_* and *σ_s_* = 1/*R_s_* as a function of temperature are plotted in Figure 7d–f for each sample, and the inserts show the ideality factor and the average barrier voltage *ϕ_B_*. These results are consistent with the hopping conduction of the Equation (2) determined for the LC-CNTs, in which *σ_p_* tends to zero and represents the conductance through the nanotubes.

Additionally, the ideality factor increases as the temperature drops since the TE mechanism tends to vanish. This behavior is reported for Schottky junctions in the case of tunneling through the contact barrier [57]. Moreover, the rise of *ϕ_B_* when increasing the temperature in Schottky contacts is related to the temperature-activated charge carriers across the interface. The electrons at low temperatures can surmount the barriers through tunneling, and when the temperature increases, the carriers gain enough energy to reach the higher barrier through thermionic emission. Consequently, the obtained *ϕ_B_* will raise with the increase in temperature and bias voltage [58,59]. These results confirm that the chosen model is satisfactory with the morphology and nature of all samples.

Finally, the existence of Schottky barriers between the LC-CNTs and Si reinforce that the mechanism of gas sensing in the devices is through the permeability of the analytes at the heterojunction. This mechanism is explained to the chemisorption of the analytes on the interface, the layers, or the contact barriers that changes the local density of charge carriers and induces a relatively large difference in the electrical measurements [60,61,62]. Even though all samples have the same sensing mechanism, there is an optimal CNT morphology in such a way that the conductivity is propitious to transport the charge. For instance, the thinner LC-CNTs have high resistance, and the electrical response cannot be readout. On the other hand, when the tubes have thicker walls, they present lower resistance, and the parallel equivalent circuit suppresses the Schottky contact effect.

Although there are devices with higher gas sensitivity than what is presented here, it is possible to propose the decoration of the internal walls of the LC-CNTs with nanoparticles to enhance their performance [63]. This opens the possibility to adapt the samples in an attractive humidity sensor or multimode analyte analyzer by reading out the electronic response [64]. The highly-order constitution of the nanotubes with perfectly perpendicular orientation opens the possibility to establish a robust platform to develop on-chip devices [65].

## 4. Conclusions

The fabrication of LC-CNTs arrays with controllable dimensions on Si substrates was successfully achieved using PAMs as templates. The low crystallinity of CNTs was established by Raman spectroscopy and HRTEM. This condition was confirmed by the electrical transport characterization, which shows that the CNTs exhibit a localization of electronic states which depend on their wall thickness, causing 3D-VRH to be the dominant electric transport mechanism. As the wall thickness increases, the electronic wave functions are more delocalized and emerge a metallic transport mechanism parallel to the 3D-VRH.

The arrays containing LC-CNTs with wall thickness in the range of 0.4 to 1.1 nm, exhibit a strong dependence of their resistance as a function of H_2_ and C_2_H_2_ concentrations in an Ar atmosphere. The most representative array was the one containing LC-CNTs with 0.7 nm wall thickness, and it exhibits a maximum resistive response of 5% for C_2_H_2_ and 1% for H_2_ analytes at 5% of concentration. This sample shows a fast response in the gas detection (few seconds) and a short recovery time (few minutes). The origin of this sensing response is related to the existence of a Schottky junction between LC-CNTs and Si. This heterojunction seems to be responsible for gases to permeate and disturb the electrical transport in a specific wall thickness range of LC-CNTs. By engineering the junctions, it is possible to optimize the gas sensing response of these arrays.

## Figures and Tables

**Table 1:** Fitting parameters for the experimental conductance of Figure 4.

*w* (nm)	*T*_0_ (K)	*G_h_* (S/m^2^)	*G_m_* (S/m^2^)
0.7 ± 0.4	8.6 ± 0.2 × 10^7^	6.0 ± 0.9 × 10^9^	1.0 ± 0.1 × 10^−4^
1.1 ± 0.4	7.1 ± 0.2 × 10^7^	1.0 ± 0.1 × 10^10^	1.1 ± 0.1 × 10^−2^
1.9 ± 0.4	1.8 ± 0.1 × 10^6^	2.4 ± 0.3 × 10^4^	3.0 ± 0.3 × 10^−2^
3.2 ± 0.4	2.4 ± 0.1 × 10^5^	1.6 ± 0.1 × 10^3^	2.1 ± 0.2 × 10^−2^

**Table 2:** Maximum resistive response measuring under exposure to 5% of H_2_ and C_2_H_2_ concentration in Ar atmosphere for several LC-CNTs devices.

*w* (nm)	H_2_ Max. Resp. (%)	C_2_H_2_ Max. Resp. (%)	Conductance/Area (S/m^2^)
0.3 ± 0.4	0	0	2.41 ± 0.02 × 10^−3^
0.4 ± 0.4	2.7 ± 0.1	5.2 ± 0.1	1.62 ± 0.01 × 10^−1^
0.7 ± 0.4	1.0 ± 0.2	5.7 ± 0.1	1.94 ± 0.02 × 10^−1^
1.1 ± 0.4	0.4 ± 0.2	1.3 ± 0.2	2.00 ± 0.02 × 10^0^
1.9 ± 0.4	0	2.2 ± 0.2	2.90 ± 0.03 × 10^0^
3.2 ± 0.4	0	0	6.83 ± 0.07 × 10^0^

**Table 3:** Fitted parameters of I–V curves as a function of wall thickness.

*w* (nm)	*n*	*R_p_* (Ω)	*R_s_* (Ω)	*I_s_* (nA)	*SC* (%)	*A* (m^2^)	*ϕ_B_* (eV)
0.4 ± 0.4	1.09 ± 0.01	11226 ± 171	1093 ± 16	0.11 ± 0.18	>91	5.5 × 10^−8^	0.34 ± 0.04
0.7 ± 0.4	1.00 ± 0.01	5019 ± 106	1110 ± 28	0.13 ± 0.54	>82	9.0 × 10^−8^	0.35 ± 0.11
1.1 ± 0.4	1.05 ± 0.01	3891 ± 36	872 ± 14	0.13 ± 0.26	>82	1.4 × 10^−7^	0.36 ± 0.05
